# Granulomatous nephropathy: have you thought about genetics?

**DOI:** 10.1007/s00467-025-06741-1

**Published:** 2025-03-18

**Authors:** Enzo Vedrine, Lucie Bessenay, Carole Philipponnet, Marine Dancer, Aurelia Bertholet-Thomas

**Affiliations:** 1 Service de Néphrologie, Centre de Référence Des Maladies Rénales Rares, Filières de Santé Maladies Rares ORKID Et ERKNet, Hôpital Édouard-Herriot, Hospices Civils de Lyon, Lyon, France; 2 Department of Pediatric Nephrology, Centre Hospitalier Universitaire de Clermont-Ferrand, Clermont-Ferrand, France; 3 Nephrology, Dialysis and Transplantation Department, University Hospital, Clermont-Ferrand, France; 4 Consortium Auragen, LBMMS Auragen, Lyon, France; 5 Pediatric Nephrology - Rheumatology and Dermatology Unit, Centre de Référence Des Maladies Rénales Rares, Filières de Santé Maladies Rares ORKID Et ERKNet, Hôpital Femme Mère Enfant, Hospices Civils de Lyon, Bron, France

**Keywords:** Granulomatous nephropathy, Nephronophthisis, Monogenic nephropathy, Pangenomic analysis

## Abstract

We report here the case of a 16-year-old girl with chronic kidney disease, where biopsy revealed tubulointerstitial nephropathy with granulomas. Initial treatments included immunosuppressive therapy unless genetic testing with exome sequencing identified nephronophthisis due to a homozygous deletion of the *NPHP1* gene, marking a unique instance of granulomatous nephropathy related to nephronophthisis. With severe kidney damage, her function has not recovered, necessitating peritoneal dialysis and transplantation. This case highlights the need to consider nephronophthisis in inflammatory interstitial and granulomatous nephropathy, especially when it appears severe and early in life. In addition, it underscores the importance of genetic testing for accurate diagnosis and management in pediatric nephropathies.

## Introduction

Granulomatous nephropathies are most often due to renal sarcoidosis (RS), a rare manifestation of a multisystem inflammatory disease characterized by the formation of non-caseating granulomas. While sarcoidosis primarily affects the lungs and lymphatic system, it can also involve kidneys, leading to interstitial nephritis (with or without granuloma), altered calcium metabolism and potentially to chronic kidney disease (CKD). Histopathological features of RS include multiple well-formed, non-caseating granulomas with multinucleated giant cells, predominantly in the cortex. While these granulomas are typically non-necrotizing, necrosis may occasionally be observed, and granulomas may also be absent. Additionally, classical features of tubulointerstitial nephritis are observed with mononucleate infiltrate and chronic patterns such as interstitial fibrosis, tubular atrophy can also be present [1].

Granulomas are a hallmark of chronic inflammation and are, of course, not specific to sarcoidosis. They can be caused by various conditions: infectious diseases such as tuberculosis, bacterial infections, fungal infections, and parasitic infections but also non-infectious causes such as autoimmune diseases like tubulointerstitial nephritis with uveitis (TINU) syndrome or Sjogren disease or vasculitis, as well as exposure to foreign substances and medication [1]. Distinguishing between these conditions is essential for appropriate diagnosis and treatment.

We report an unusual case of granulomatous nephropathy in a young girl followed in our pediatric nephrology unit. The diagnosis was made through a pangenomic test, highlighting the importance of a systematic etiological investigation for every patient.

## Case presentation

A 16-year-old girl was admitted to our pediatric nephrology unit in Lyon, France, in November 2023 for evaluation of stage 5 CKD (creatinine 1648 µmol/L, urea 76 mmol/L) with secondary hyperparathyroidism and severe anemia (hemoglobin 72 g/L) without vitamin or iron deficiency. Acute management included initiation of hemodialysis and intravenous antihypertensive treatment in the intensive care unit. Ultrasound revealed kidneys of preserved size and significant low molecular weight proteinuria (200 mg/mmol) was noted, matching with severe tubulointerstitial nephritis.

**Fig. 1:**
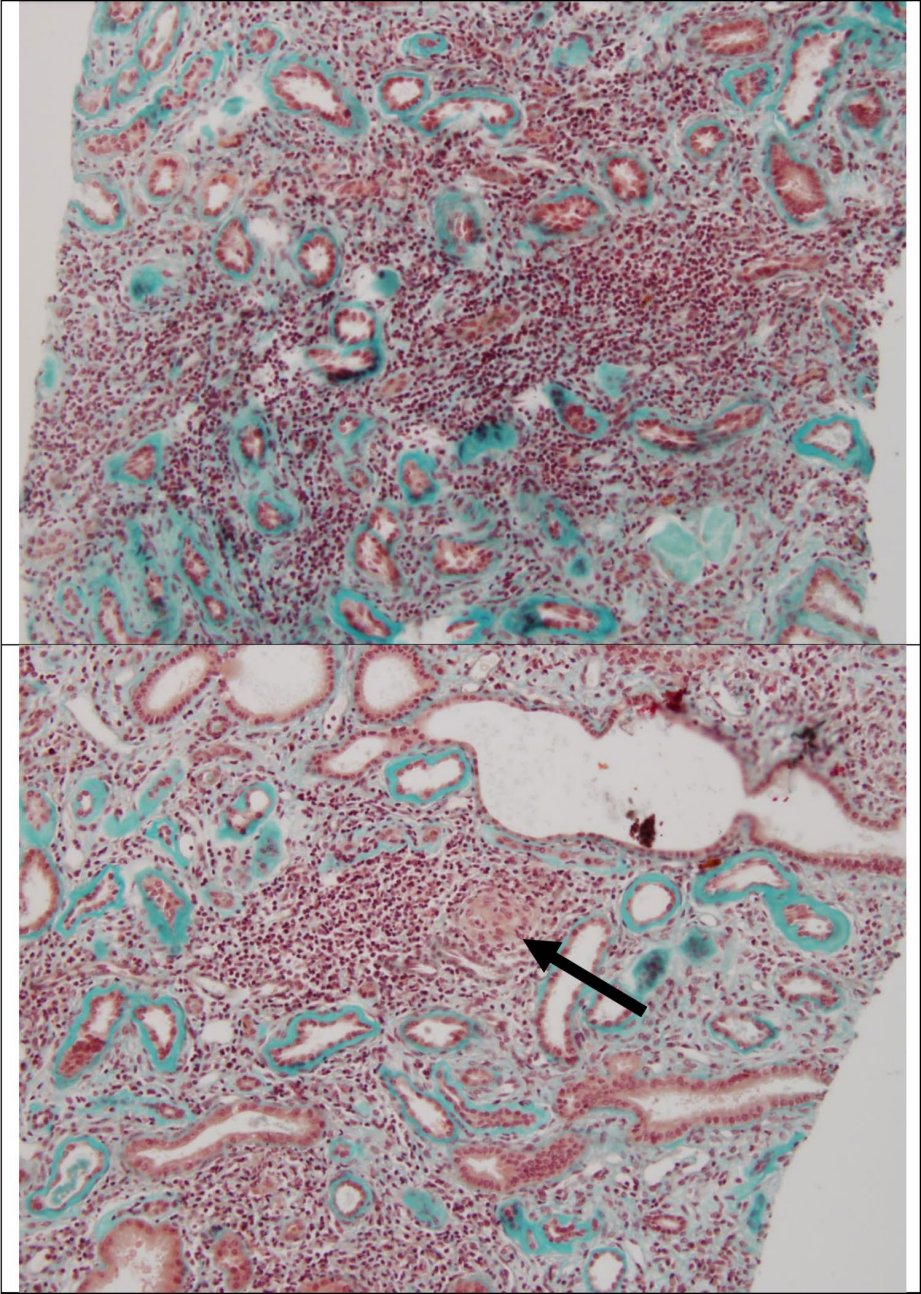
Histopathological results of the kidney biopsy. Histological section, stained with Masson’s trichrome. Dense interstitial inflammatory infiltrate within a fibrous zone, absence of healthy parenchyma. The tubes show marked thickening of the tubular vitreous. Another section also stained with Masson’s trichrome, again showing a dense interstitial inflammatory infiltrate within a very fibrous parenchymal zone. This time, we note the presence of a granulomatous reaction with giant cells (dark arrow). Tubular vitreous is very thick, with the tubes sometimes appearing dilated and cystic

Histological findings, represented in Fig. 1, from the kidney biopsy revealed tubulointerstitial nephropathy with inflammatory lesions, including mononuclear infiltrates and non-necrotizing granulomas. Extensive interstitial fibrosis and advanced tubular atrophy and marked thickening of their basement membrane indicated advanced CKD. Glomeruli showed no proliferative lesions, although some exhibited focal segmental glomerulosclerosis changes. Immunofluorescence and immunohistochemistry for IgG4 were negative.

A comprehensive workup for granulomatosis was initiated. Clinical examination revealed no other manifestations of granulomatosis (e.g., hypercalcemia, hypercalciuria, or elevated ACE) and laboratory tests showed no relevant signs. Neither CT scan nor 18F-PET scan revealed extra-renal manifestations. Investigations for intracellular pathogens were negative, as were hematological exams for malignant conditions. Considering severe granulomatous nephropathy, immunosuppressive treatment with methylprednisolone pulses was initiated, followed by oral prednisone and mycophenolate mofetil.

Considering the young age of the patient, lack of improvement of kidney function with immunosuppressive therapy, and the probably chronic status of the kidney disease, genetic analysis (exome sequencing) was performed and led to the diagnosis of nephronophthisis. Homozygous Copy Number Variation with deletion of the *NPHP1* gene was observed. The patient’s parents were unrelated but *NPHP1* deletion is the most common observed variation in this gene. Confirmation was achieved through multiplex ligation-dependent probe amplification. Retro phenotyping revealed no extra-renal involvement such as liver disease, retinopathy, or neurological abnormalities. As expected, our patient did not recover kidney function and was transitioned to peritoneal dialysis before transplantation in September 2024.

## Discussion

This is the first reported case of granulomatous nephropathy associated with nephronophthisis, a leading cause of genetic kidney failure in children. Nephronophthisis is classified among ciliopathies, which are disorders of the primary cilium, an organelle essential for cellular homeostasis, differentiation, and development [2]. Numerous genes are involved in ciliopathies with kidney implications, leading to various phenotypes, including polycystic kidney disease, Bardet-Biedl syndrome, and different forms of nephronophthisis like Joubert and Senior-Loken syndromes. *NPHP1* is the most frequently involved gene in isolated late-onset nephronophthisis [3]. Interstitial inflammation has been well documented in biopsies from patients with nephronophthisis, often involving neutrophils, macrophages, and T cells regulated by a specific set of inflammatory mediators [4]. While inflammation is a dominant feature of nephronophthisis, a form as inflammatory as our patient’s has never been reported. *NPHP1* deletion seems to be the main variation in NPHP genes, diagnosed in 20% of patients with nephronophthisis with a genetic diagnosis. The isolated kidney disease presented in this case is atypical for RS, as over 90% of RS cases have extra-renal involvement [1] and rarely manifest at CKD stage 5. The severity of kidney damage was disproportionate to the extent of cellular infiltration, indicating an underlying etiology that warranted genetic investigation. Identifying the genetic cause led to cessation of immunosuppressive therapy, avoiding potential adverse drug effects [5].

This finding also provided clarity to the patient and her family regarding the absence of kidney function recovery and reassured them about the low recurrence risk post-transplant. Nephronophthisis, like many other genetic kidney diseases (e.g., ciliopathies, podocytopathies, cystic nephropathies…), does not recur on the graft. Establishing the etiological diagnosis of unknown kidney disease ruled out pathologies likely to recur, such as complement disorders (atypical hemolytic uremic syndrome and C3 glomerulonephritis) and metabolic disorders, such as primary hyperoxaluria [5]. Additionally, it enabled early screening for kidney damage in the patient’s younger sister.

It is debatable whether the patient suffered from two different diseases. However, since sarcoidosis and nephronophthisis are both rare diseases, the interstitial inflammation usually seen in nephronophthisis, together with the absence of other damage consistent with a possible autoinflammatory disease, do not support this hypothesis. Moreover, the patient is today at 15 months post transplantation without any recurrence sign or extra-renal manifestation.

Nephronophthisis should be considered a potential cause of granulomatous nephropathy, particularly in severe and early-onset cases. Genetic testing remains essential in the etiological evaluation of pediatric nephropathies, especially when the diagnosis is uncertain. The paradigm is shifting, with expanding indications for genetic evaluation, allowing clinicians to implement precise and personalized management.

## Summary

### What is new?

- First description of granulomatous histopathologic form of nephronophthisis.
- Underscores the place of genetic testing as a cornerstone of evaluation of pediatric nephropathies.
- Personal medicine and avoiding unnecessary immunosuppressive treatment.
